# An unusual cause of simultaneous common bile and pancreatic duct dilation

**DOI:** 10.1093/gastro/gou032

**Published:** 2014-06-17

**Authors:** Puneet Chhabra, Surinder Singh Rana, Vishal Sharma, Ravi Sharma, Rajesh Gupta, Deepak Kumar Bhasin

**Affiliations:** ^1^Department of Gastroenterology, Postgraduate Institute of Medical Education and Research (PGIMER), Chandigarh, India and; ^2^Department of Surgery, Postgraduate Institute of Medical Education and Research (PGIMER), Chandigarh, India

**Keywords:** afferent loop syndrome, pylorus preserving pancreaticoduodenectomy

## Abstract

Simultaneous dilation of both the common bile duct and the pancreatic duct (double-duct sign) is usually because of ampullary or pancreatic tumours. Here we report an unusual cause of double-duct dilation; we describe the case of a 49-year-old female who developed afferent loop syndrome after pylorus-preserving pancreaticoduodenectomy: cross-sectional imaging of the abdomen revealed a double-duct sign.

## INTRODUCTION

Whipple’s surgery is associated with a number of complications, such as pancreatic fistula, post-surgical haemorrhage, porto-mesenteric venous, pancreatitis, hepatic infarction, delayed gastric emptying, and anastomotic strictures [1]. Afferent loop syndrome (ALS) is defined as obstruction of the small bowel, at a point proximal to the site of anastomosis with the stomach, with accumulation of bile acid and pancreatic juice in the afferent loop. It is a very rare complication of Whipple’s surgery and the causes of obstruction commonly described are torsion of the loop, compression by adhesions, volvulus, internal hernias, recurrence of the malignant process, and anastomotic site stricture [2]. We describe a middle-aged female who, after pylorus-preserving pancreaticoduodenectomy, developed ALS due to kinking of the bowel and in whom cross-sectional imaging of the abdomen revealed a double-duct sign.

## CASE PRESENTATION

**Figure 1:**
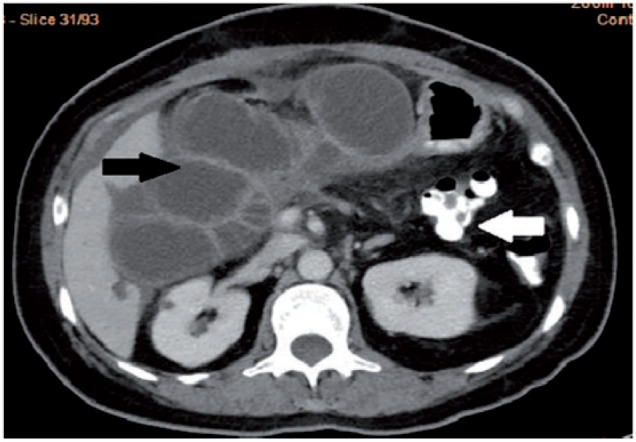
CECT of the abdomen, showing dilated, fluid-filled afferent loop (black arrow) and normal efferent loop with oral contrast (white arrow).

**Figure 2:**
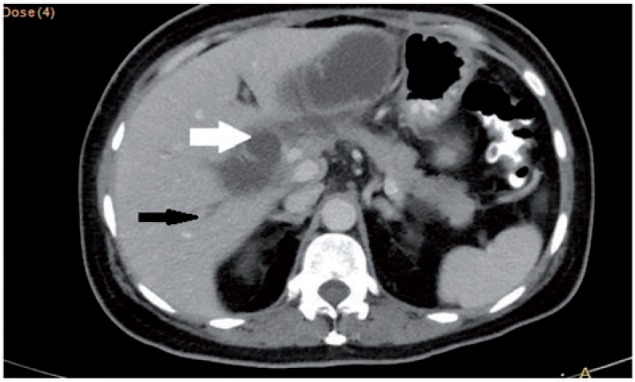
CECT of the abdomen: dilated common bile duct (white arrow) with dilated intrahepatic biliary radicles (black arrow).

**Figure 3:**
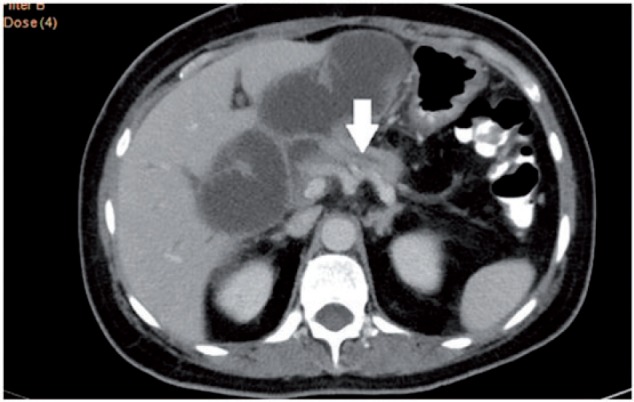
CECT of the abdomen: dilated pancreatic duct.

**Figure 4:**
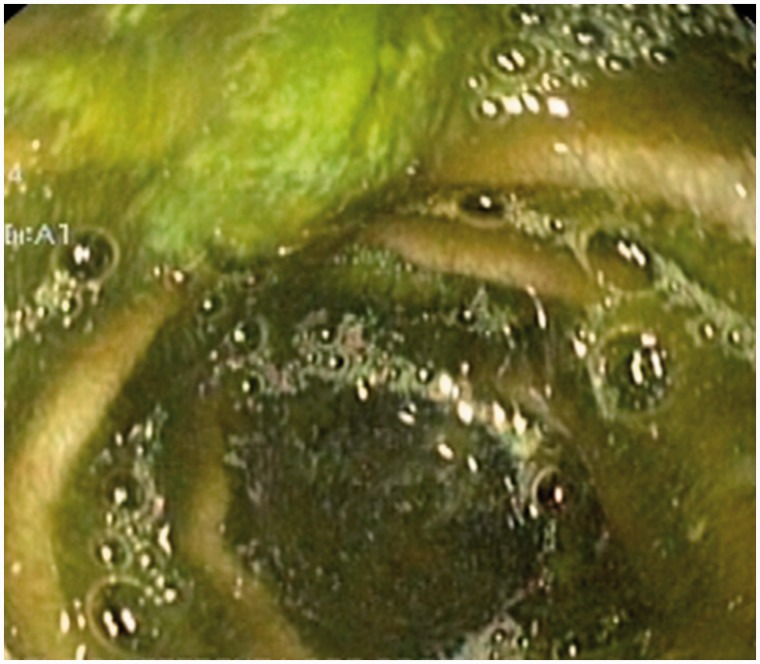
Dilated afferent loop beyond the obstruction filled with bilious residue.

**Figure 5:**
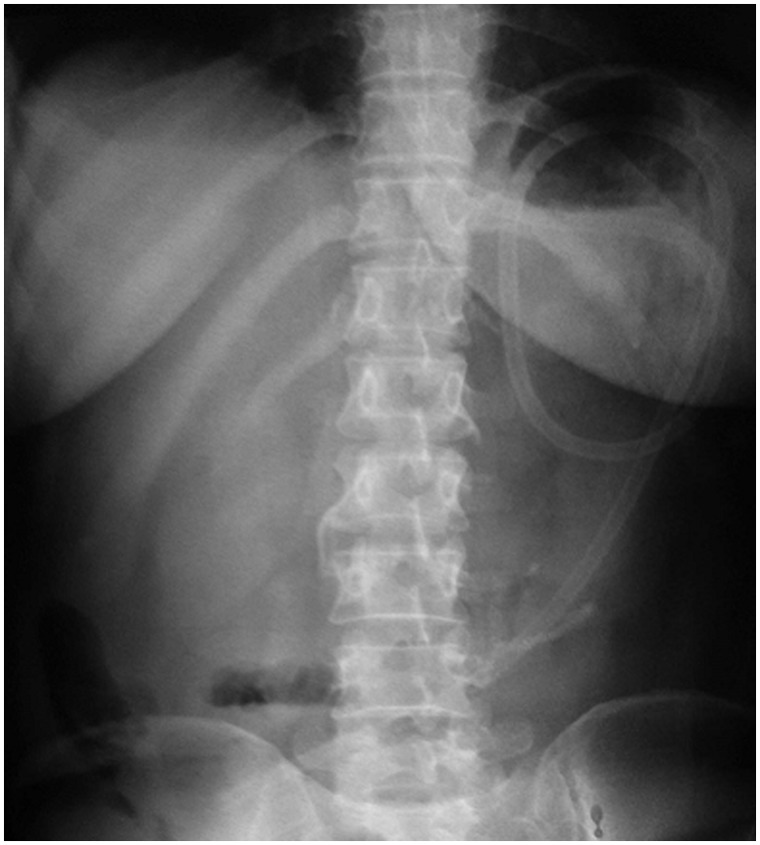
NJ tube in the afferent loop.

A 49-year-old female presented with upper abdominal pain, accompanied with abdominal distension. She had undergone pylorus-preserving pancreaticoduodenectomy for pancreatic neuroendocrine tumour three months previously. The patient had tachycardia and icterus, and abdominal examination revealed tenderness in the upper abdomen. Serum amylase, bilirubin and alkaline phosphatase levels were elevated. Contrast-enhanced computerized tomography (CECT) of the abdomen revealed a markedly dilated afferent loop of gastro-jejunal (GJ) anastomosis (Figure 1) with normal efferent loops. The intra-hepatic biliary radicals, common bile duct and pancreatic duct were also dilated (Figures 2 and 3). Gastroscopy revealed an obstruction in the afferent loop, 2 cm beyond the anastomosis, and there was marked difficulty in progressing beyond this narrowing. The afferent loop beyond the obstruction was markedly dilated and filled with bilious fluid (Figure 4). No mass or ulcerations were observed at or near the obstruction and there was a sharp bend. Around one litre of bilious fluid was sucked out and a naso-jejunal (NJ) tube was put in place for further decompression (Figure 5). Following this, the patients improved markedly and the NJ tube was removed after two weeks. The patient is currently asymptomatic after one month of follow-up.

## DISCUSSION

ALS is most commonly described following Billroth Type 2 surgery, which is carried out for complicated peptic ulcer disease [2]. Afferent loop syndrome can have an acute or chronic presentation. The acute presentation is with a complete obstruction and usually occurs within first few weeks of surgery. It is characterized with severe upper abdominal pain and vomiting, with a rapidly deteriorating clinical course [3]. On the other hand, chronic ALS usually occurs several months or years after surgery and is associated with partial obstruction of the afferent loop. It usually presents as vague abdominal pain and distension that subsides following bilious vomiting [3]. In rare cases, the increased pressure in the afferent loop can cause dilation of the bile and pancreatic ducts, which can present as cholangitis or rarely as pancreatitis [2–5].

Early diagnosis is very important in preventing life-threatening complications such as afferent loop perforation [6]. ALS can be investigated using barium studies, abdominal ultrasound, endoscopy, CECT and magnetic resonance cholangiopancreatography (MRCP). Plain X-rays of the abdomen or routine laboratory tests are rarely helpful [3]. Barium meal follow-through can help in diagnosis by showing non-filling of the afferent loop but about 20% of normal afferent loops are also not filled, limiting the utility of this method in the diagnosis of ALS [6,7]. Abdominal ultrasonography—an initial investigation done in a patient with abdominal pain—may show a large and dilated bowel loop in the mid-abdomen, lying anterior to the abdominal aorta [8]. However, it may be difficult to differentiate it from a pseudocyst on ultrasound and CECT of the abdomen is therefore the investigation of choice for useful for determining the site, degree, and cause of ALS. The technique shows a dilated fluid-filled afferent loop coming out of the gall bladder fossa, crossing the mid-line in the anatomical location of the third part of the duodenum [4, 7, 9]. Multidetector-row computed tomography (MDCT) has been shown to improve the diagnostic capability of CT for diagnosis of ALS, and fluid-filled C-shaped afferent loop (C-loop appearance) on the coronal plane—in combination with *valvulae conniventes* projecting into the lumen (keyboard sign)—has been shown to be the most common MDCT features of ALS [9]. MRCP can also be used to demonstrate afferent loop obstruction with dilation of both the common bile duct and pancreatic duct [10].

Management of afferent loop syndrome includes endoscopic, percutaneous or surgical approaches. Conservative management has no role except on very rare occasions, as in chronic obstruction, where emptying of the afferent loop contents into the stomach may relieve obstruction, or in patients with disseminated malignancy. Surgical correction has been the treatment of choice but radiological or endoscopic treatment has also been successfully performed in selected patients [3, 9]. Endoscopic decompression can be carried out under direct vision or by placing stents through the trans-gastric route into the dilated afferent loop under endoscopic ultrasound (EUS) guidance [11, 12]. Transoral catheters have been placed endoscopically for diagnostic and therapeutic purposes, as was done in our case [13]. This provides temporary relief of symptoms and the patients usually need surgery for a more definitive cure; however, patients who have disseminated malignancy or who are unfit for surgery may need minimally invasive endoscopic approaches. Patients with benign diseases may benefit from the insertion of double-pigtail stents across the obstruction, whereas patients with malignant disease can be treated with endoscopic placement of self-expanding metallic stents (SEMS) [14, 15]. The percutaneous route has also been exploited; more so for transhepatic biliary drainage when ALS presents as cholangitis [16].

**Conflict of interest:** none declared.

## References

[1] Raman SP, Horton KM, Cameron JL (2013). CT after pancreaticoduodenectomy: spectrum of normal findings and complications. AJR Am J Roentgenol.

[2] Woodfield CA, Levine MS (2005). The post-operative stomach. Eur J Radiol.

[3] Kim JK, Park CH, Huh JH (2011). Endoscopic management of afferent loop syndrome after a pylorus preserving pancreatoduodenecotomy presenting with obstructive jaundice and ascending cholangitis. Clin Endosc.

[4] Warrier RK, Steinheber FU (1979). Afferent Loop Obstruction presenting as obstructive jaundice. Dig Dis Sci.

[5] Conter RL, Converse JO, Mcgarrity TJ (1990). Afferent Loop Obstruction presenting as acute pancreatitis and pseudocyst. Surgery.

[6] Cartanese C, Campanella G, Milano E (2013). Enterolith causing acute afferent loop syndrome after Billroth II gastrectomy: a case report. G Chir.

[7] Wise SW (2000). Afferent Loop Syndrome. Radiology.

[8] Lee DH, Lim JH, Ko YT (1991). Afferent loop syndrome: sonographic findings in seven cases. AJR Am J Roentgenol.

[9] Juan YH, Yu CY, Hsu HH (2011). Using multidetector-row CT for the diagnosis of afferent loop syndrome following gastroenterostomy reconstruction. Yonsei Med J.

[10] McKee JD, Raju GP, Edelman RR (1997). MR cholangiopancreatography (MRCP) in diagnosis of afferent loop syndrome presenting as cholangitis. Dig Dis Sci.

[11] De martino C, Caiazzo P, Albano M (2012). Acute afferent loop obstruction treated by endoscopic decompression. Case report and review of literature. Ann Ital Chir.

[12] Bamba S, Shiomi H, Fujiyama Y (2013). Afferent loop syndrome successfully treated by endoscopic ultrasound-guided transgastric drainage. Dig Endosc.

[13] Paulsen O, Skjennald A, Osnes M (1987). An endoscopic drainage procedure for afferent loop occlusion. Gastrointest Endosc.

[14] Burdick JS, Garza AA, Magee DJ (2002). Endoscopic management of afferent loop syndrome of malignant etiology. Gastrointest Endosc.

[15] Hosokawa I, Kato A, Shimizu H (2012). Percutaneous transhepatic metallic stent insertion for malignant afferent loop obstruction following pancreaticoduodenectomy: a case report. J Med Case Rep.

[16] Lee LI, Teplick SK, Haskin PH (1987). Refractory afferent loop problems: percutaneous transhepatic management of two cases. Radiology.

